# Automatic detection and delineation of pediatric gliomas on combined [^18^F]FET PET and MRI

**DOI:** 10.3389/fnume.2022.960820

**Published:** 2022-08-24

**Authors:** Claes Nøhr Ladefoged, Otto Mølby Henriksen, René Mathiasen, Kjeld Schmiegelow, Flemming Littrup Andersen, Liselotte Højgaard, Lise Borgwardt, Ian Law, Lisbeth Marner

**Affiliations:** ^1^Department of Clinical Physiology and Nuclear Medicine, Rigshospitalet, University of Copenhagen, Copenhagen, Denmark; ^2^Department of Pediatrics and Adolescent Medicine, Rigshospitalet, University of Copenhagen, Copenhagen, Denmark; ^3^Department of Clinical Medicine, University of Copenhagen, Copenhagen, Denmark; ^4^Department of Clinical Physiology and Nuclear Medicine, Bispebjerg Hospital, University of Copenhagen, Copenhagen, Denmark

**Keywords:** deep learning, decision support, convolutional neural network, brain tumor, CNS tumor, children, neuro-oncology

## Abstract

**Introduction:**

Brain and central nervous system (CNS) tumors are the second most common cancer type in children and adolescents. Positron emission tomography (PET) imaging with radiolabeled amino acids visualizes the amino acid uptake in brain tumor cells compared with the healthy brain tissue, which provides additional information over magnetic resonance imaging (MRI) for differential diagnosis, treatment planning, and the differentiation of tumor relapse from treatment-related changes. However, tumor delineation is a time-consuming task subject to inter-rater variability. We propose a deep learning method for the automatic delineation of O-(2-[^18^F]fluoroethyl)-l-tyrosine ([^18^F]FET PET) pediatric CNS tumors.

**Methods:**

A total of 109 [^18^F]FET PET and MRI scans from 66 pediatric patients with manually delineated reference were included. We trained an artificial neural network (ANN) for automatic delineation and compared its performance against the manual reference on delineation accuracy and subsequent clinical metric accuracy. For clinical metrics, we extracted the biological tumor volume (BTV) and tumor-to-background mean and max (TBR_mean_ and TBR_max_).

**Results:**

The ANN produced high tumor overlap (median dice-similarity coefficient [DSC] of 0.93). The clinical metrics extracted with the manual reference and the ANN were highly correlated (*r* ≥ 0.99). The spatial location of TBR_max_ was identical in almost all cases (96%). The ANN and the manual reference produced similar changes in the clinical metrics between baseline and follow-up scans.

**Conclusion:**

The proposed ANN achieved high concordance with the manual reference and may be an important tool for decision aid, limiting inter-reader variance and improving longitudinal evaluation in clinical routine, and for future multicenter studies of pediatric CNS tumors.

## Introduction

Brain and central nervous system (CNS) tumors are the second most common cancer type (21%) in children and adolescents (1), and CNS tumors have poor prognosis and severe late effects (1–3). The standard imaging method is magnetic resonance imaging (MRI), which offers high sensitivity for the detection of brain and spinal cord tumors. However, in the post-treatment setting, MRI may be challenged in the differentiation between treatment-related changes and tumor tissue, resulting in lower specificity (4–9).

Positron emission tomography (PET) imaging with radiolabeled amino acids visualizes upregulated active transport in brain tumor cells compared with the healthy brain tissue, providing additional information on metabolic properties. The most commonly used tracers are O-(2-[^18^F]fluoroethyl)-l-tyrosine ([^18^F]FET), [^11^C-methyl]-methionine ([^11^C]MET), and 3,4-dihydroxy-6-[^18^F]-fluoro-l-phenylalanine ([^18^F]FDOPA), which are recommended in neuro-oncology imaging guidelines for children and adults for differential diagnosis, treatment planning, and the differentiation of tumor relapse from treatment-related changes (10–12). Pathological amino acid accumulation measured using the tumor-to-brain ratio (TBR) on static [^18^F]FET PET images can be used to differentiate neoplastic and non-neoplastic tissues, provide tumor grading, and estimate the biological tumor volume (BTV) (11), which is prognostic for overall survival in post-resection glioblastoma multiforme in adults (13). An analysis of the tracer uptake dynamics using a time-activity curve (TAC) extracted from 40 to 50 min dynamic [^18^F]FET PET imaging further increases diagnostic accuracy (14). Image-driven biomarkers extracted through radiomic features have recently shown potential for the differentiation of IDH-wildtype from IDH-mutant genes (15), as well as for progression-free survival and overall survival prognosis (16).

The results from studies performed on adult CNS tumors might not be extrapolated to pediatric patients as pediatric CNS tumors show clinical and biological features that are distinct from adult tumors. However, [^18^F]FET PET can add valuable information for clinical decision-making (5, 17), and the combination of MRI and [^18^F]FET PET was recently shown to improve the low specificity of MRI alone (1.00 compared with 0.48) while maintaining a high sensitivity across 64 pediatric patients with 83 treated lesions (5).

Pediatric CNS cancers are rare with high heterogeneity in tumor types, which warrants multicenter studies to validate the abovementioned findings in a larger cohort of patients. This requires a reproducible delineation of the tumor tissue, which is a time-consuming task potentially subject to intraobserver and interobserver variability (18). With the advancement of artificial intelligence (AI), several tools exist for fully-automatic segmentation of biological images, such as the statistical inference method (16), the traditional machine learning techniques (19), and the generic deep-learning-based algorithms (20, 21). One such deep-learning method was trained using 27 [^18^F]FET PET/CT adult subjects and achieved a high dice-similarity coefficient (DSC) of 0.79 across 11 test subjects with a manually delineated reference (22). To the best of our knowledge, none of these algorithms have been tested on a pediatric cohort of patients with [^18^F]FET PET CNS tumor.

Here, we aimed to develop and train an automatic segmentation algorithm based on a large cohort of single-institutional dataset of pediatric patients with CNS tumor to delineate [^18^F]FET PET lesions and determine clinically relevant metrics to perform a large number of multicenter trails using automatic delineation avoiding the time consuming human-drawn delineations.

## Materials and methods

Deep learning models require large amounts of training data to be accurate. In this study, we hypothesized that our deep learning model would be more accurate if it was trained using transfer learning from a larger cohort of adult patients, despite differences in anatomy and tumor biology. Thus, we created two datasets for this study. The first dataset consists of adult patients with neuro-oncology examined with [^18^F]FET PET and serves as a training set for the initial model. The second dataset consists of pediatric patients serving for training and validation.

### Patients

For the pediatric dataset, we included children and adolescents with suspicion or diagnosis of primary CNS tumors before the age of 18 years examined with [^18^F]FET PET between February 2015 and January 2019. The patients were part of a larger study of [^18^F]FET PET/MRI in primary CNS tumors in children and adolescents approved by the regional ethical committee (ID: H-6-2014-095) and registered at Clinicaltrials.gov (NCT03402425) and acquired with written informed consent for participation from parents. We refer to the original study for detailed patient characteristics (5). The dataset included scans performed at initial diagnosis, before or after surgery, at the response assessment or at suspected relapse. Compared with the original study, we limited the cohort to patients with tumors in the brain, thereby excluding 10 patients with tumors only located in the spinal cord and patients without [^18^F]FET active tumor >0.1 ml. A total of 109 scans from 66 patients were thus included. The median age was 10.6 years (ranged 0.1–19.5 years). A total of 10 pediatric patients had a minimum of three scans performed and were used for a longitudinal evaluation (Section Analysis).

For the adult dataset, the department archive was screened for clinical patients who underwent surgery for histologically proven glioma or intracerebral metastasis and had simultaneously acquired [^18^F]FET PET and MRI using our PET/MRI system between October 2018 and January 2021, and 233 [^18^F]FET PET/MRI scans were identified that had a [^18^F]FET active tumor >1 ml. No other inclusion or exclusion criteria were imposed. This study was performed according to the Danish legislation as a quality assurance study with permission from the hospital administration. The need for explicit written consent was not required according to regulations since the study operated exclusively on anonymized retrospective data. To comply with legal requirements, all data were fully anonymized upon collection from the clinical archives in compliance with the General Data Protection Regulation (GDPR).

### Imaging protocol

For the pediatric dataset, PET and MRI scans were performed as previously described (23). PET and MRI were performed on our hybrid PET/MRI system (3T Biograph mMR, Siemens, Erlangen, Germany) to reduce the number of scanning procedures (*n* = 86 scans) or alternatively in two sessions where the PET scan was performed using a PET/CT system (Biograph TruePoint, Siemens, Erlangen, Germany) and the MRI scan was performed on a 3T MRI system (*n* = 23 scans). The PET scan was performed according to international guidelines (11) and included a 40-min dynamic acquisition commenced at the same time as the intravenous injection of the tracer (3 MBq/kg), from which a summed image was generated by reconstructing the last 20 min of the acquisition. Attenuation correction of the PET data acquired on the PET/MRI system was done using a co-registered same-day low-dose CT image. The MRI protocol was in accordance with the international guidelines (24, 25) and included, among other sequences, a post-contrast 3D isotropic T1-weighted MPRAGE (CT1w).

For the adult dataset, [^18^F]FET PET/MRI was performed as previously described (26). The MRI protocol included a pre-contrast 3D isotropic T1-weighted MPRAGE.

### Image processing

Manual delineation of metabolically active tissue was performed as previously described for both datasets (5, 27). In brief, the delineation was performed in Mirada (Mirada Medical, Oxford, UK) by placing an auto-contour defining tumor tissue at a threshold above 1.6 of the mean uptake in a background region of interest (ROI) placed in healthy appearing gray and white matter in a contralateral hemisphere to the tumor. Extratumoral areas with high [^18^F]FET uptake, e.g., vascular structures, pineal body, and skin, were identified on either the MR or PET image and were not included. The delineation was performed by a nuclear medicine specialist experienced in pediatric neuro-oncology (LM) and adult neuro-oncology (IL) for the pediatric and adult datasets, respectively.

Image preprocessing of the pediatric dataset involved brain extraction followed by the resampling of MRI images to PET resolution. Brain extraction was performed using the HD-BET (28) on the CT1w images, but since this method removes the lower part of the medulla oblongata that can contain CNS tumors, we merged the brain mask with a medulla mask found by rigidly aligning the MNI template (29) to the patient's CT1w MRI (reg_aladin and nifty-reg). We dilated the combined brain and medulla masks for 20 iterations to compensate for any registration or brain extraction inaccuracies. Image preprocessing of the adult data only included brain extraction using the HD-BET.

We trained an artificial neural network (ANN) to perform the automatic segmentation using the U-Net architecture (30). We utilized nnU-Net to train the ANN, which is an end-to-end solution for data preprocessing and network training (21). We used default nnU-Net settings for training a 3D full resolution network with PET and CT1w images as input and our ground truth label as target. Details regarding the preprocessing, network architecture, and hyperparameters are shown in Supplementary Table 1. We trained a total of three nnU-Net architectures: (1) trained with only adult data, (2) trained with only pediatric data, and (3) trained with pediatric data with transfer learning from the adult network. The network with transfer learning was trained with a reduced initial learning rate of 10^−4^ but otherwise identical hyperparameters to the other two architectures.

We trained the pediatric models using a 10-fold cross-validation analysis to obtain delineations for all subjects. Each network was therefore, on average, trained with 99 scans using the nnU-Nets internal five-fold cross-validation, leaving 11 scans for testing in each fold.

### Analysis

We had three main objectives. The first was to evaluate the accuracy of the trained models for CNS tumor delineation against the manually delineated volumes using the calculated dice-similarity coefficient (DSC) metrics for each model output against the manual reference. A DSC of 1 is achieved when there is a complete overlap between the reference and ANN delineation. We compared the statistical difference between the models using the Wilcoxon signed-rank test in R version 3.6.1. The superior model was used for the subsequent analysis. The sensitivity, specificity, positive predictive value (PPV), and negative predictive value (NPV) at the voxel level were calculated.

The second objective was to evaluate the clinically relevant PET-metrics extracted using the delineated volumes on a patient-by-patient basis. These metrics include the most commonly used semi-quantitative clinical metrics in the diagnostic workflow. Similar to previous studies (27, 31), we measured the mean and maximum tumor-to-background ratio (TBR_mean_ and TBR_max_) within each biological tumor volume (BTV) as well as the size of the BTV. These metrics are commonly used as a criterion to discriminate between active tumor tissue and reactive changes. The spatial location of peak TBR_max_ was compared as this is often used for biopsy target planning (32–35). Furthermore, we extracted the time-activity-curve (TAC) for each patient and computed the time-to-peak (TTP) in minutes of the full 40-min TAC. Such kinetic imaging parameters might be prognostic of overall survival (36), and may add valuable information for the clinical decision-making in pediatric patients (17). For each TAC, we assigned one of the following curve patterns following (37): constantly increasing without identifiable peak uptake (pattern I); peak at the midway point (>20 min) followed by a plateau or a small descent (pattern II); and early peak (<20 min) followed by a constant descent (pattern III).

The third objective was to evaluate the performance of the automatic segmentation in longitudinal datasets. We evaluated the robustness of the ANN method over time by calculating TBR_mean_, TBR_max_, and BTV for each baseline and follow-up examination, respectively, and compared each to the reference manual delineation.

## Results

**Figure 1:**
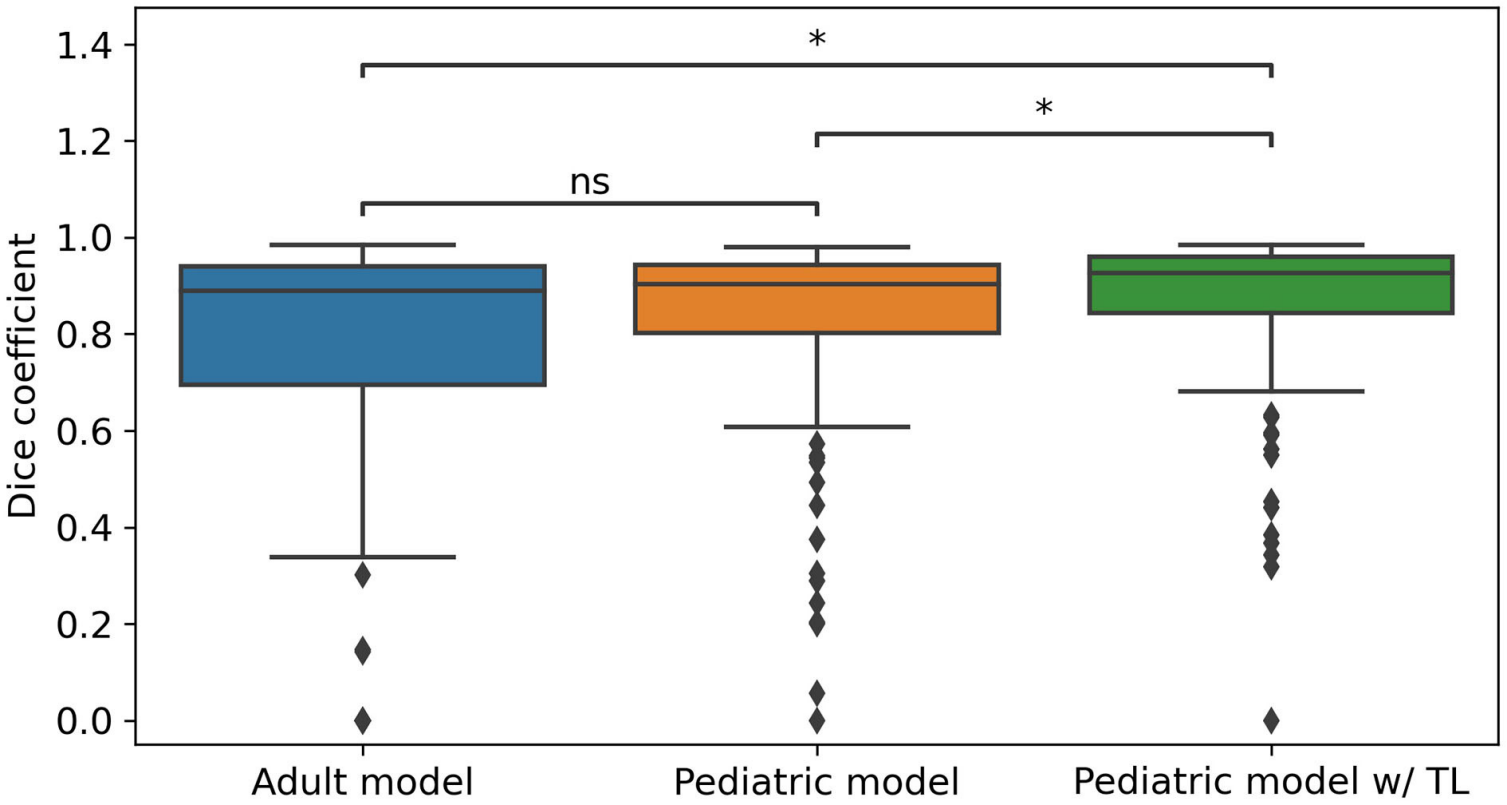
Dice-similarity coefficient (DSC) comparison of the three trained models for all pediatric scans (*n* = 109). The adult model was trained using only adult fluoroethyl-L-tyrosine (FET) PET/MRI data (*n* = 233) and directly applied to the pediatric data. The pediatric model was trained using only pediatric data, and the pediatric model w/TL was trained using the same data but transfer learned (TL) from the adult model. A Wilcoxon signed-rank test was performed for statistically significant differences where *ns* indicates not significant and * indicates significance with a *p*-value < 0.05.

**Figure 2:**
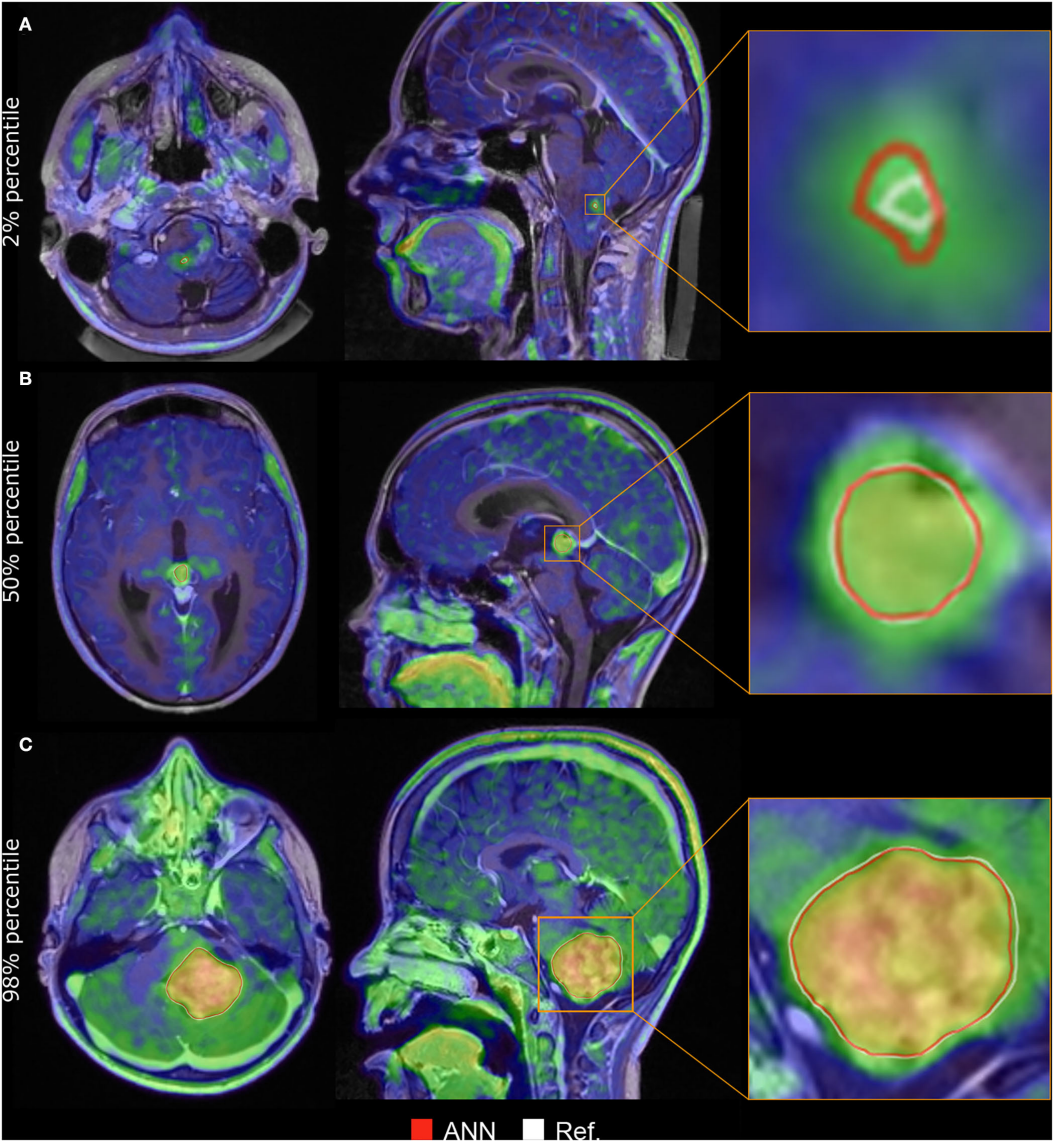
Delineation performance for three cases chosen at 2% **(A)**, 50% **(B)**, and 98% **(C)** of the dice-similarity coefficient (DSC). The white line represents the manual delineation (Ref.), while the red line is the artificial neural network (ANN) delineation. **(A)** A 17-year-old boy with a pilocytic astrocytoma, DSC = 0.32. **(B)** A 14-year-old boy with a germinoma, DSC = 0.93. **(C)** A 7-year-old boy with a pilocytic astrocytoma, DSC = 0.98. All three were scanned at primary diagnosis.

**Figure 3:**
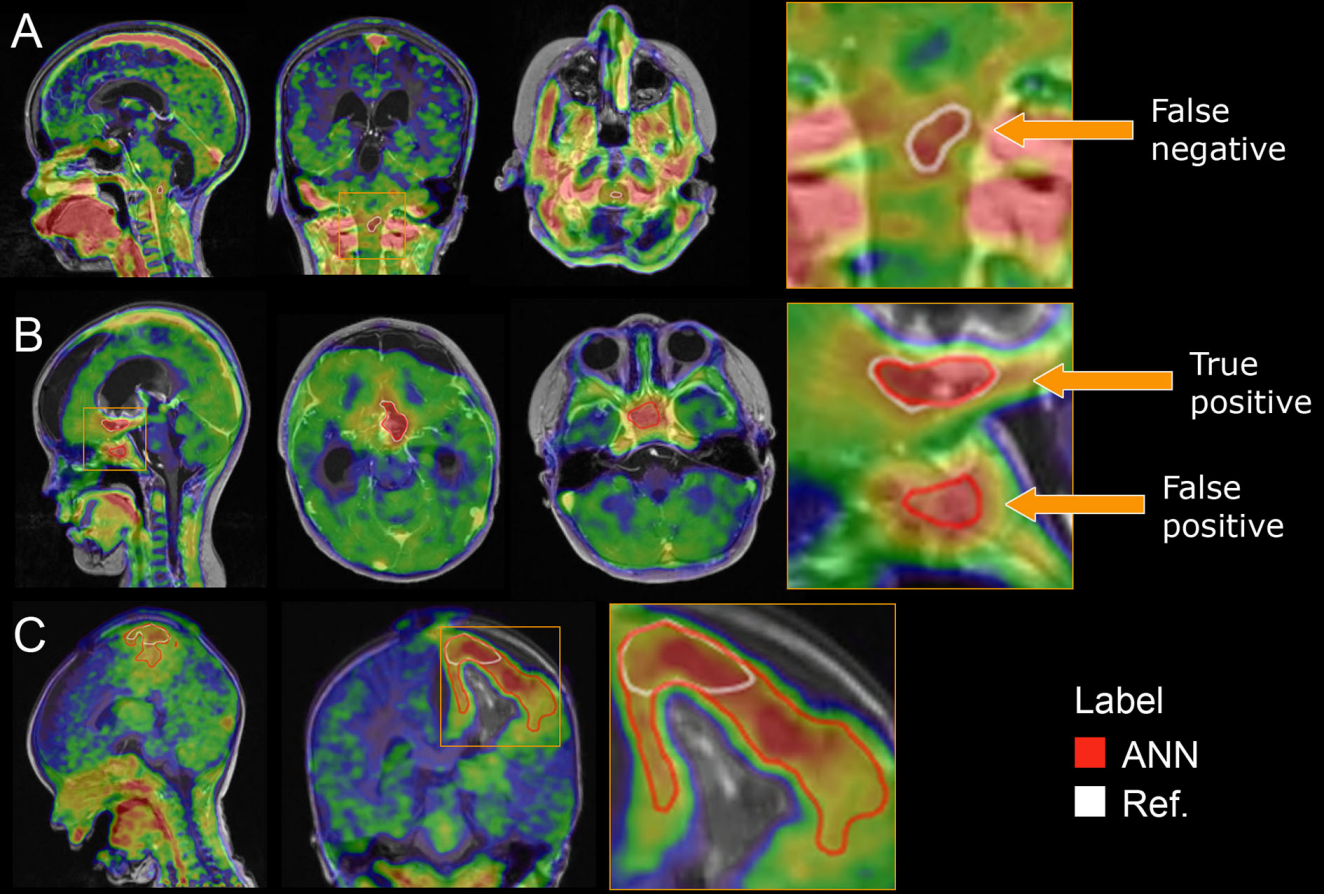
Outlier cases. **(A)** The FET-positive area in the upper medulla failed to be delineated by the ANN, DSC = 0. **(B)** The ANN correctly delineates the FET-positive area in the bottom of the operation cavity but incorrectly delineates a region in the pituitary region, DSC = 0.59. **(C)** The ANN correctly detects the tumor but overestimates the area (22 ml) compared with the reference (6 ml) as the cortical pattern of uptake reflects reactive changes after surgery and is not included in the manual reference delineation, DSC = 0.37.

The ANNs yielded a median DSC of 0.89 (95% CI: 0.83–0.94) using the adult model, 0.90 (0.86–0.94) with the pediatric model, and 0.93 (0.89–0.96) with the pediatric model with transfer learning from the adult model (Figure 1). There was no statistical difference between the adult and pediatric models without transfer learning (the Wilcoxon signed-rank test, *p* > 0.05, 95% CI −0.04 to 0.01). The pediatric model with transfer learning was significantly better than both the adult models (*p* < 0.05, 95% CI −0.06 to −0.01) and the pediatric model without transfer learning (*p* < 0.05, 95% CI −0.04 to −0.0003). Thus, the pediatric model with transfer learning was chosen for the remaining evaluation and will be referred to as the ANN model. Figure 2 shows the worst, average, and best segmentation performance of the ANN for representative cases selected using the 2, 50, and 98% for DSC. The ANN model accurately detected the CNS tumor in most cases (83% with DSC above 0.8) but failed to locate the tumor in one case (Figure 3A).

The evaluation at the voxel level (Table 1) resulted in a sensitivity of 0.90 (95% *CI*, 87.2–92.8%), a specificity of 1.0 (99.9%−100%), a PPV of 0.85 (81.8–88.3%), and a NPV of 1.0 (99.9%−100%).

**Table 1:** Confusion matrix at the voxel level across the set of 109 scans restricted to voxels within the brain and medulla mask extracted during pre-processing.

**Predicted/truth**	**True**	**False**
True	1,178,883	89,657
False	77,127	171,502,554

**Figure 4:**
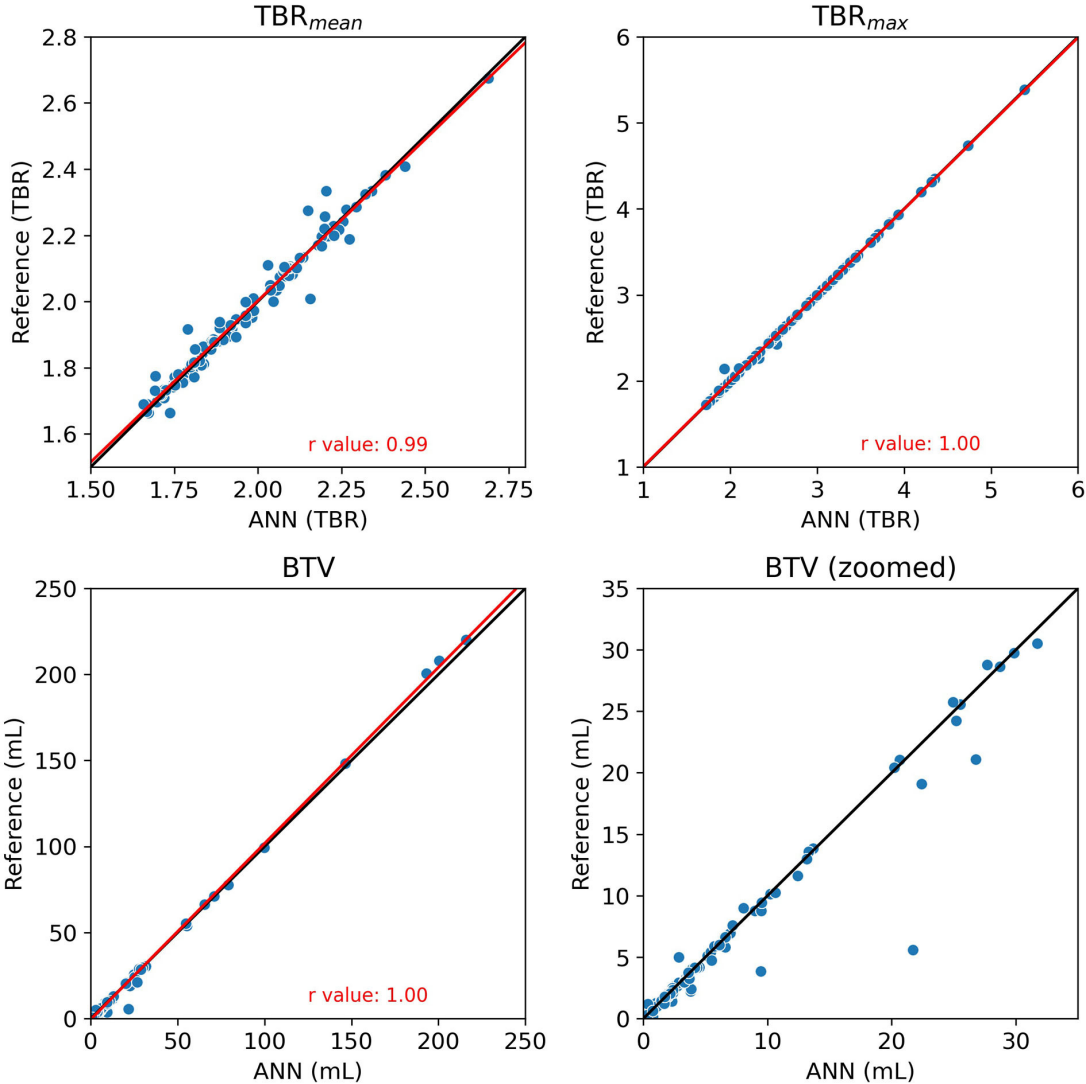
The comparison of the clinical metrics TBR_mean_, TBR_max_, and BTV extracted with the ANN (*x*-axis) and the reference manual delineation (*y*-axis). The biological tumor volume (BTV) is also plotted for the 0–35 ml range for a better comparison of the smaller lesions. The black line is the identity line and the red line is a linear fit to the points with the *r*-values indicated in the figure.

The clinical evaluation is shown in Figure 4. Using the ANN-extracted TBR metrics correlates well with the reference-extracted metrics (*r* ≥ 0.99). The ANN and reference delineation resulted in similar BTV measurements, albeit with some outliers for the smallest (<10 ml) tumors. Examples of the largest outliers are shown in Figures 3B,C. The spatial location of peak TBR_max_ was identical in 96% of the cases and was 19, 31, and 38 mm in the remaining three cases with an annotated tumor in both reference and ANN. The TTP calculated from the dynamic PET data was identical in 85% of the cases and within ± one frame in 99% of the cases. The assigned TAC patterns I–III were congruent in all but one case, where a false positive lesion found in the pituitary region (Figure 3B) overestimated the peak at the initial frames.

**Figure 5:**
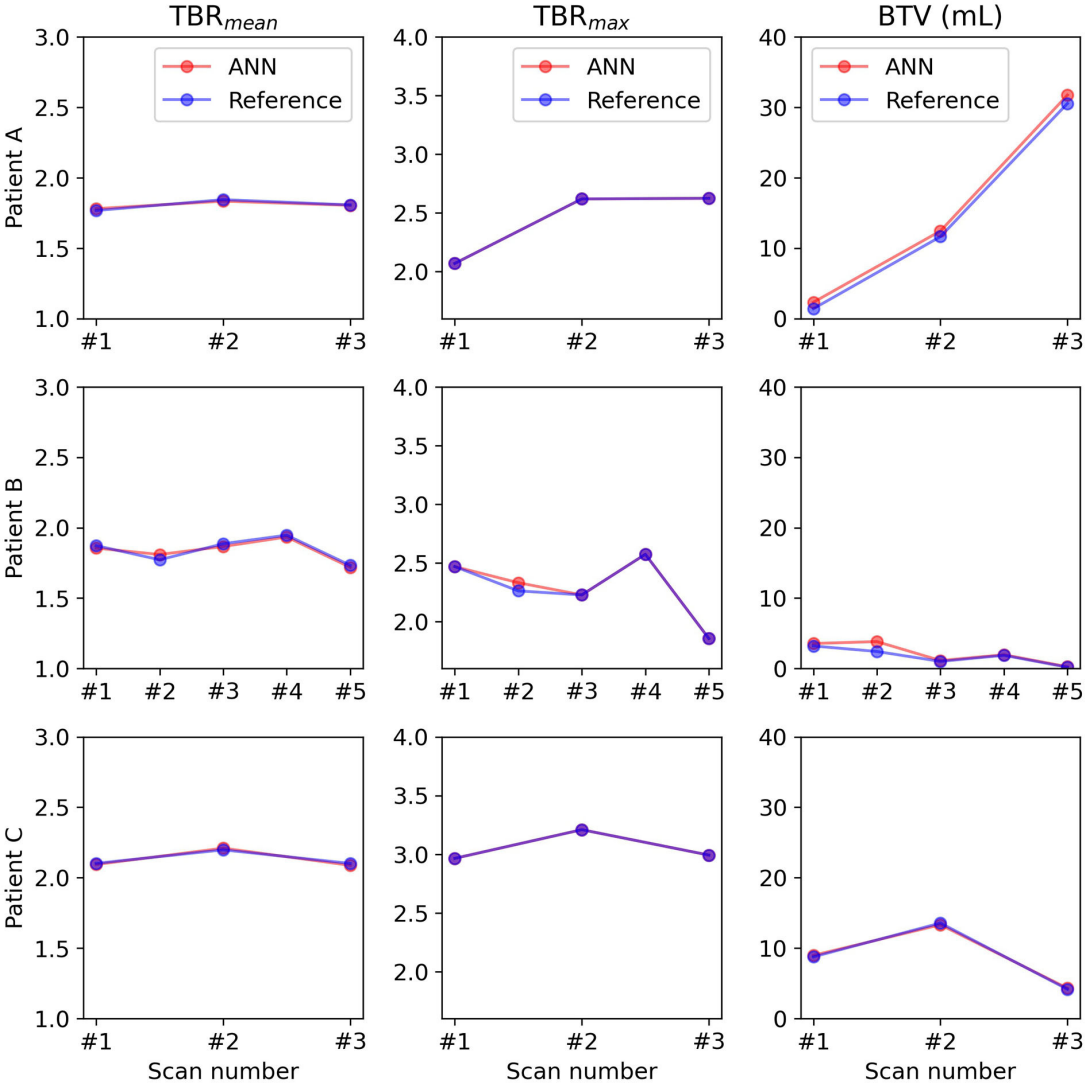
Longitudinal evaluation with clinical metrics for three representative patients. # indicates the number.

The longitudinal evaluation of TBR_mean_, TBR_max_, and BTV are shown in Figure 5 for three representative subjects. The direction and magnitude of the change between baseline, and each follow-up scan was congruent for each clinical metric between reference and ANN delineation for all 10 patients.

## Discussion

This study evaluated the feasibility of a fully automated detection and delineation method for pediatric CNS tumors from [^18^F]FET PET and MRI. We proposed and trained an ANN that was pre-trained on adult subjects with brain gliomas (*n* = 233) and found an increased performance over training on the pediatric subjects alone. This is, to the best of our knowledge, the first study for automatic tumor delineation on pediatric patients scanned with [^18^F]FET PET and MRI.

Most of the lesions were correctly found and accurately delineated by the ANN, resulting in clinical metrics (TBR_mean_, TBR_max_, and BTV) with similar values regardless of the delineation method. The TBR_max_ was the metric most robust with only two scans notably deviating from the identity line. The difference was in one case caused by the ANN not delineating a FET-positive hotspot at the edge of a surgery cavity that was otherwise correctly delineated, and in the other by the ANN incorrectly delineating a vessel (Figure 3A). The high correlation for the TBR_max_ metric and an agreement of peak TBR_max_ location indicates that, while absolute differences might exist between manual and automatic delineated BTV, the spatial peak is correctly found. This further allows for accurate automatic placement of a circular or spherical volume-of-interest (VOI) at TBR_max_, which is an alternative to full tumor delineation (37). As expected, the variation was higher for TBR_mean_ values since the metric is affected by even minor differences in the delineation boundary. These findings are important since the TBR metrics can be used clinically to separate the reactive tissue from the tumor tissue and since implementing the ANN method in daily clinical routine may save time and increase inter-reader reproducibility.

Similar BTV was found with the reference and ANN delineation methods. As expected, the largest relative errors were found for the small tumors (BTV <10 ml) and could often be attributed to a 1–2 voxel difference at the border (as shown in, e.g., Figure 2A). We observed a few cases where the ANN incorrectly delineated non-tumor tissue, e.g., a region in the pituitary region (Figure 3B). Such errors might be attributed to the large heterogeneity of tumor types, location, and patient age in the training cohort. The inclusion of more patients might reduce the false positive delineations. Further use of kinetic parameters, such as TTP, might also help with the differentiation.

Central nervous system tumor delineation in pediatric subjects requires knowledge about viable tumor location and likely growth pattern. Figure 3C illustrates an example of the latter where the ANN overestimates the tumor area, mimicking the performance of a naïve TBR thresholding. For this patient, the reference delineation is a result of a subjective decision of the boundary location as the area of [^18^F]FET uptake above the TBR threshold represents a typical cortical reactive pattern after recent surgery (23). An ANN is not capable of mimicking such subjective decisions when the cases are underrepresented in the dataset. Thus, the manual inspection and correction of the delineations is a requirement, in particular, if they are applied for clinical routine use. The inclusion of more datasets with typical treatment changes may improve performance.

The clinical metrics are further important for the assessment of treatment response (10). We found nearly identical metrics (TBR and BTV) for the ANN when compared with the reference delineation in the longitudinal dataset. This result indicates that the use of our ANN is feasible in follow-up studies as any error in BTV appears to be present in both the baseline and subsequent follow-up examinations. Further studies with a larger cohort of longitudinal datasets are required to confirm this finding.

The main limitation of our study is the low number of patients in the test set since pediatric CNS tumors are rare and highly heterogeneous. Even though the number of patients exceeds similar studies for [^18^F]FET PET brain tumor delineation (22, 36), the number of patients in each sub-category is low when divided into tumor type and patient age. Future inclusion of patients might therefore further improve the results and minimize the false positive/negative tumors.

Comparison with alternative methods is challenging since no automatic delineation method for pediatric CNS tumors currently exists. Blanc-Durand et al. (22) proposed a deep learning method for the segmentation of gliomas in [^18^F]FET PET/CT scans of adult patients and achieved a mean DSC of 0.79 (22). The mean DSC in our dataset was comparatively higher (0.86), suggesting the state-of-the-art performance of our method.

The fast inference time for the ANN (<1 min) suits well in the clinical routine, as manual delineation is a time-consuming task often associated with high inter-reader variance. The full tumor delineation allows for the use of [^18^F]FET PET alongside MRI for machine learning, deep learning, and radiomic models for tumor classification and prognosis (38).

## Conclusion

We implemented and validated an ANN for the automatic delineation of pediatric CNS tumors. The method achieved high concordance with the manual reference and performed on par with the state-of-the-art [^18^F]FET PET delineation of brain gliomas in adult subjects. The method allows for automatic delineation with either no or limited user intervention, which provides volumetric measurements of biological tumor volume as well as clinically relevant metrics for differentiating tumor tissue from treatment-related reactive changes. The ANN may be an important tool for decision aid, to limit inter-reader variance and improve longitudinal evaluation in clinical routine, and for future multicenter studies of pediatric CNS tumors.

## Data availability statement

The data analyzed in this study is subject to the following licenses/restrictions. The data contain patient identifiable information. Requests to access these datasets should be directed at: lisbeth.marner@regionh.dk.

## Ethics statement

The studies involving human participants were reviewed and approved by National Committee on Health Research Ethics. Written informed consent to participate in this study was provided by the participants' legal guardian/next of kin. Written informed consent was not obtained from the minor(s)' legal guardian/next of kin for the publication of any potentially identifiable images or data included in this article.

## Author contributions

CL designed the method, did the data analysis, and prepared the manuscript. OH, FA, LH, and LB aided in the data analysis and revised and approved the manuscript. RM, KS, IL, and LM aided in data acquisition, aided in data analysis, and revised and approved the manuscript. All authors contributed to the article and approved the submitted version.

## Funding

This work is part of the Danish Nation-Wide Research Program Childhood Oncology Network Targeting Research, Organization & Life expectancy (CONTROL) and supported by the Danish Cancer Society (R-257-A14720) and the Danish Childhood Cancer Foundation (2019-5934 and 2020-5769).

## Conflict of interest

The authors declare that the research was conducted in the absence of any commercial or financial relationships that could be construed as a potential conflict of interest.

## Publisher's note

All claims expressed in this article are solely those of the authors and do not necessarily represent those of their affiliated organizations, or those of the publisher, the editors and the reviewers. Any product that may be evaluated in this article, or claim that may be made by its manufacturer, is not guaranteed or endorsed by the publisher.
