# Supplementary material for: Automatic detection and delineation of pediatric gliomas on combined [18F]FET PET and MRI
Source: Front Nucl Med. 2022 Aug 24;2:960820. doi: 10.3389/fnume.2022.960820 (PMC11440972; doi:10.3389/fnume.2022.960820)
Supplement: Supplementary file 1 [file Table_1.DOCX]

Supplementary Material

**Supplementary Table 1:** nn-UNet hyper parameters for pediatric model transfer learned from adult cohort.

| **Data preprocessing** | | |
| --- | --- | --- |
| Input voxel spacing | | 2.03x0.83x0.83 mm^3^ |
| Median shape after pre-processing | | 71x205x165 voxels |
| Patch size | | 64x192x160 voxels |
| Normalization schemes: | | |
|  | PET | CT |
|  | MRI | nonCT |
| **Network details** | | |
| Network | | 3d_fullres |
| Trainer | | nnUNetTrainerV2* |
| Initial learning rate | | 10^-4^ |
| Number of base features | | 32 |
| Batch size | | 2 |
| Max epochs | | 1000 |
| Number of batches per epoch | | 250 |
| net_conv_kernel_sizes | | [[1,3,3], [3,3,3], [3,3,3], [3,3,3], [3,3,3], [3,3,3]] |
| net_num_pool_op_kernel_sizes | | [[1,2,2], [2,2,2], [2,2,2], [2,2,2], [2,2,2]] |
| num_pool_per_axis | | [4,5,5] |
| **Data augmentation settings** | | |
| do_elastic | | False |
| elastic_deform_alpha | | (0.0, 900.0) |
| elastic_deform_sigma | | (9.0, 13.0) |
| p_eldef | | 0.2 |
| do_scaling | | True |
| scale_range | | (0.7, 1.4) |
| independent_scale_factor_for_each_axis | | False |
| p_independent_scale_per_axis | | 1 |
| p_scale | | 0.2 |
| do_rotation | | True |
| rotation_x | | (-0.52, 0.52) |
| rotation_y | | (-0.52, 0.52) |
| rotation_z | | (-0.52, 0.52) |
| rotation_p_per_axis | | 1 |
| p_rot | | 0.2 |
| random_crop | | False |
| random_crop_dist_to_border | | None |
| do_gamma | | True |
| gamma_retain_stats | | True |
| gamma_range | | (0.7, 1.5) |
| p_gamma | | 0.3 |
| do_mirror | | True |
| mirror_axes | | (0, 1, 2) |
| dummy_2D | | False |
| mask_was_used_for_normalization | | OrderedDict([(0, False), (1, True)]) |
| border_mode_data | | constant |
| all_segmentation_labels | | None |
| move_last_seg_chanel_to_data | | False |
| cascade_do_cascade_augmentations | | False |
| cascade_random_binary_transform_p | | 0.4 |
| cascade_random_binary_transform_p_per_label | | 1 |
| cascade_random_binary_transform_size | | (1, 8) |
| cascade_remove_conn_comp_p | | 0.2 |
| cascade_remove_conn_comp_max_size_percent_threshold | | 0.15 |
| cascade_remove_conn_comp_fill_with_other_class_p | | 0.0 |
| do_additive_brightness | | False |
| additive_brightness_p_per_sample | | 0.15 |
| additive_brightness_p_per_channel | | 0.5 |
| additive_brightness_mu | | 0.0 |
| additive_brightness_sigma | | 0.1 |
| num_cached_per_thread | | 2 |
| patch_size_for_spatialtransform | | [64, 192, 160] |

* nnUNetTrainerV2 was extended for transfer learning by initializing with a lower learning rate.
